# Effect of storage duration on carprofen concentration measurements in dog plasma

**DOI:** 10.1002/vms3.1215

**Published:** 2023-07-20

**Authors:** James R. Shuttleworth, Kristen N. Behrens, Morgan R. Biggo, Rikki L. Horne, Sherry Cox, Jeffrey Lakritz, Selena Tinga

**Affiliations:** ^1^ The Ohio State University College of Veterinary Medicine Columbus Ohio USA; ^2^ University of Tennessee College of Veterinary Medicine Knoxville Tennessee USA; ^3^ Present address: Cornell University College of Veterinary Medicine Ithaca NY USA

**Keywords:** carprofen, cryostorage, HPLC, plasma, storage

## Abstract

**Background:**

Storage of samples may be necessary prior to testing drug levels in certain study designs; however, the effect of storage duration on measured drug levels is not known for all drugs.

**Objectives:**

The objective of this study was to evaluate the stability of carprofen in canine plasma when stored at −80°C for 6 months.

**Methods:**

Six healthy dogs were enrolled (1–10 years old, 17–35 kg) and received compounded carprofen at 2.2 mg/kg orally every 12 h for 2 days. On the third day, blood was collected immediately before the morning dose (trough), then 1 and 6 h after the dose (sampling timepoint). Whole blood was immediately centrifuged, and plasma was stored at −80°C. Plasma carprofen concentration was measured at day 2, week 2 and then monthly for 6 months using reversed‐phase high‐performance liquid chromatography. The measured carprofen concentrations were analysed statistically using a linear mixed effects model.

**Results:**

There was no effect of storage time over 6 months (*p* = 0.891) on measured carprofen levels. Although there was an effect of sampling timepoint (0, 1 and 6 h) (*p* < 0.001), the interaction between storage timepoint and sampling timepoint was not statistically significant (*p* = 1).

**Conclusions:**

Carprofen‐laden canine plasma samples can be stored for up to 6 months before analysis with no degradation in carprofen concentrations expected.

## Graphical abstract

The objective of this study was to evaluate the stability of carprofen in canine plasma when stored at −80°C for 6 months. Healthy dogs received carprofen (2.2 mg/kg orally every 12 h) for 2 days, then blood was drawn and plasma carprofen concentration was measured multiple times over 6 months. There was no effect of storage time on measured carprofen concentrations (*p* = 0.891): carprofen‐laden canine plasma samples can be stored for up to 6 months at −80°C.

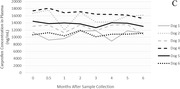

## 1 INTRODUCTION

Carprofen,[6‐chloro‐∝‐methyl‐9*H*‐carbazole‐2‐acetic acid, C₁₅H₁₂ClNO₂], is a non‐steroidal anti‐inflammatory drug (NSAID) which plays a crucial role in managing acute soft tissue injuries, orthopedic pain and chronic inflammation in many animal species. By inhibiting cyclooxygenase (COX) enzymes 1 and 2, NSAIDs decrease the release of pro‐inflammatory mediators, thereby reducing swelling, heat, pain and redness (Messenger et al., 2016).

The pharmacokinetics of carprofen have been previously published for several animal species. These studies used plasma that was stored between −20°C and −80°C until the time of analysis, but the authors do not mention storage duration or its effects on plasma carprofen concentrations (Budsberg et al., 2020; Kjaergaard et al., 2018; Morris et al., 2020). One study reported that samples were stored for up to 2 months prior to carprofen level testing but did not report any validation for the chosen storage duration (Lascelles et al., 1998). Sample storage conditions are a relevant consideration for study design where accurate drug levels are needed. Many veterinary clinical trials have ongoing enrolment, and thus blood samples may be collected over many months. If there is no degradation over time, samples could be stored then shipped to the testing laboratory in batches, which would provide consistent laboratory conditions (i.e. no effects of cleaning and calibration) and would minimize shipping costs. Alternatively, if degradation does occur with cryostorage, individual samples should be sent for testing as they are collected. The objective of this study was to determine whether plasma storage at −80°C influenced plasma carprofen concentrations at the time of analysis. The authors hypothesized there would be no change in plasma carprofen concentrations after 6 months.

## 2 MATERIALS AND METHODS

The study design was adapted from published guidelines (Gómez‐Rioja et al., 2019) on determining sampling stability and was approved by the University's Institutional Animal Care and Use Committee. We recruited six dogs that were healthy with no regularly administered medications other than ectoparasite and heartworm preventatives (Riviere et al., 2011). The dogs were not receiving steroids or NSAIDs in the 30 days before the study or any other medications in the 5 days before the study. Health was confirmed within 10 days of drug administration using physical examinations, complete blood counts, blood chemistries and urinalyses. Carprofen capsules were prepared using manufactured carprofen caplets (Carprovet (carprofen) caplets; Dechra). The carprofen caplets were ground down to a soft, fine, homogenous powder and used to make capsules specific to each patient weight. Every patient received capsules formulated for patient‐specific 2.2 mg/kg doses. Each dog received their patient‐specific capsule by mouth every 12 h starting 2 days prior to blood sample collections. On the third day of dosing, dogs had three 16‐mL blood samples collected. The first blood sample was collected immediately before the morning dose. The remaining samples were collected 1 and 6 h after the morning dose.

After each collection, the blood samples were immediately placed into evacuated plastic tubes containing sodium heparin and placed on ice. Within minutes, the samples were centrifuged at 2000*g* for 20 min, and the plasma was separated into 0.3‐mL aliquots and stored at −80°C. The following day, eight aliquots from each blood sample were shipped on dry ice to the University of Tennessee College of Veterinary Medicine Diagnostic Laboratory. Aliquots were stored at −80°C and were analysed at day 2, week 2 and then monthly for 6 months.

Carprofen plasma analysis was conducted using reversed‐phase high‐performance liquid chromatography with fluorescent detection. The compounds were separated on an Atlantis T_3_ (4.6 × 250 mL and 5 μm) column with a 5 μm Atlantis T_3_ guard column. The mobile phase was composed of 50 mM sodium phosphate monobasic pH 5 and acetonitrile (53:47, v/v). The excitation was 301 nm, and the emission was 371 nm with a flow rate of 1 mL/min.

Carprofen plasma samples were thawed and vortex‐mixed and 100 μL of plasma was transferred to a screw top tube, and 50 μL of flurbiprofen (internal standard, 100 μg/mL) was added followed by 100 μL of 1 M HCl and 1 mL of ethyl acetate. The tubes were rocked for 10 min and then centrifuged for 20 min at 1070*g*. The organic phase was transferred to a glass tube and evaporated to dryness with nitrogen. Samples were reconstituted in 250 μL of mobile phase and 100 μL injected into chromatography system.

Standard curves for plasma analysis were prepared by fortifying untreated plasma with carprofen to produce a linear concentration range of 5–10,000 ng/mL. The lower limit of quantification during validation was 5 ng/mL. The intra‐ and inter‐assay variability ranged from 3.2% to 11%, and the average recovery for carprofen was 89%.

The measured carprofen concentrations were analysed statistically using a linear mixed effects model with fixed effects of storage timepoint, sampling timepoint and their interaction, and a random effect of animal to account for repeated measurements from each animal. The fixed effects were tested using *F* tests with the Satterthwaite approximation of the denominator degrees of freedom. The model assumptions of normality and homogeneous variance were evaluated visually using plots of the model residuals and predicted values. A *p*‐value of <0.05 was considered statistically significant.

## 3 RESULTS

Demographic information for the six dogs included in this study is listed in Table 1. Owners provided informed consent and all dogs were reported to receive the compounded carprofen on schedule per their owners. All samples were collected and processed on the same day and then shipped together and tested on the same days. Median carprofen measurements are reported in Table 2. There was no significant effect of storage duration on measured carprofen levels (*F*(2,115) = 0.41, *p* = 0.891), and the interaction between sampling timepoint and storage timepoint was not statistically significant (*F*(4,115) = 0.16, *p* = 1). The main effect of sampling timepoint (0, 1 or 6 h after dosing) was statistically significant (*F*(2,115) = 12.02), *p* < 0.001).

**TABLE 1:** Demographic information for dogs included in the study.

Dog	Breed	Sex	Age (years)	Weight (kg)
1	Mixed breed dog	FS	2	17.9
2	Mixed breed dog	MN	4	28.5
3	Belgian Malinois	FS	1	26.3
4	Greyhound	MN	8	30.6
5	Mixed breed dog	FS	3	35.0
6	Mixed breed dog	MN	10	19.0
Median			3.5	28.5

Abbreviations: FS, female spayed; MN, male neutered.

**TABLE 2:** Carprofen levels at each sampling timepoint and each storage (−80°C) timepoint for six dogs.

		Sampling timepoint		
		*t*0	*t*1	*t*6
Storage timepoint	2 days	12,214 (6730–15,860)	11,989 (5979–18,964)	13,786 (10,685–17,405)
	2 weeks	10,510 (7077–14,692)	12,608 (5170–19,060)	13,608 (9179–18,077)
	1 month	11,112 (6619–14,412)	12,343 (5132–18,352)	11,626 (9596–16,848)
	2 months	10,960 (6343–15,453)	12,233 (4572–17,596)	13,571 (11,685–17,123)
	3 months	11,044 (6000–14,806)	10,612 (6303–17,432)	12,250 (10,092–16,453)
	4 months	10,328 (6885–14,741)	9,983 (5863–19,153)	13,707 (8918–16,386)
	5 months	11,036 (6020–13,959)	12,222 (5943–18,020)	13,733 (10,847–16,175)
	6 months	10,536 (5980–14,816)	10,917 (5727–17,582)	12,087 (10,660–16,140)

*Note*: Each cell represents the median (range) carprofen level for six dogs at the indicated sampling and storage timepoint, reported in ng/mL. *t*0: immediately prior to dosing (12 h after last dose); *t*1: 1 h after dosing; *t*6: 6 h after dosing.

## 4 DISCUSSION

Unlike drugs such as gabapentin where sample stability during storage is documented, the effects of storage duration on plasma carprofen have not been reported (Tjandrawinata et al., 2014). Therefore, this study's objective was to determine the effects of cryostorage at −80°C on measured carprofen concentrations up to 6 months after sampling, and we accepted our hypothesis: There was no change in measured carprofen concentrations over 6 months of storage. The ability to store and batch samples is variably important depending on study design and pharmacology laboratory access. This may be most important for prospective studies that are dependent on clinical caseload, where animals are enrolled individually over time, and the samples must be shipped to an offsite laboratory. If shipped individually, the samples could experience variable conditions during shipping or even testing depending on factors such as the laboratory's calibration schedules or if equipment is replaced between samples. The information provided in this study should allow researchers to confidently store and batch samples for testing and therefore help to standardize test results, because storage at −80°C for up to 6 months will not affect study results.

The only factor analysed in this study that was noted to have an influence on measured carprofen concentration was blood sample collection time in relation to time of carprofen administration. Carprofen concentrations are expected to vary over time as the drug is absorbed and then excreted. Carprofen reaches peak plasma concentrations 0.5–3 h after oral administration (Clark et al., 2003). The terminal half‐life of carprofen ranges from 3.2 to 11.8 h with a mean of approximately 8 h (McKellar et al., 1990). All samples were within or slightly above the therapeutic dose range of carprofen (1000–17,000 ng/mL) at all sampling and storage timepoints (Nolan & Reid et al., 1993).

Limitations on the present study primarily include the narrow application to research methods as the study was designed to fill this gap in the literature. A larger sample size may have demonstrated a statistical change in carprofen levels with storage time; although the sample size used here is generally accepted for this type of study (Riviere et al., 2011), the statistical methodology was robust, and the *p*‐value was quite high. In addition, a 3.2%–11% variability in laboratory results for the positive control was noted. Only 89% of the drug was recovered from the positive control so exact carprofen concentration may vary slightly from the reported numbers. An additional limitation is that these data cannot be used to infer stability at other common storage temperatures such as −20°C. Further studies would be needed to assess stability under other storage conditions. Finally, we compounded the carprofen capsules in our pharmacy and these capsules were not tested for identity, quality, strength, purity or stability. However, the study was designed to analyse the effect of storage after initial plasma carprofen concentrations were measured, rather than the pharmacokinetics of carprofen, so this should not affect the conclusions of this study.

## 5 CONCLUSIONS

In conclusion, storage duration does not impact measured carprofen concentration in plasma when stored at −80°C for 6 months. Sample storage for variable durations of time up to 6 months followed by batched testing should not lead to variable degradation of carprofen and therefore should not affect measured carprofen levels in a significant manner. These results only apply to the storage of dog plasma with therapeutically relevant concentrations of carprofen and cannot be extrapolated to other drugs.

## AUTHOR CONTRIBUTIONS

*Conceptualization; data curation; formal analysis; investigation; methodology; project administration; writing (original, review and editing)*: James R. Shuttleworth and Kristen N. Behrens. *Conceptualization; methodology; resources; validation; writing (review and editing)*: Morgan R. Biggo. *Conceptualization; formal analysis; investigation; methodology; supervision; validation; writing (review and editing)*: Sherry Cox. *Conceptualization; methodology; resources; supervision; writing (review and editing)*: Jeffrey Lakritz. *Conceptualization; data curation; formal analysis; funding acquisition; investigation; methodology; project administration; resources; supervision; writing (original, review and editing)*: Selena Tinga.

## CONFLICT OF INTEREST STATEMENT

The authors have no conflicts of interest to declare.

## FUNDING INFORMATION

Funding was obtained from faculty discretionary funds.

### ANIMAL WELFARE AND ETHICS STATEMENT

The authors confirm that the ethical policies of the journal, as noted on the journal's author guidelines page, have been adhered to and the appropriate ethical review committee approval has been received (IACUC 2020A00000018). The authors confirm that they have adhered to US standards for the protection of animals used for scientific purposes.

### PEER REVIEW

The peer review history for this article is available at https://publons.com/publon/10.1002/vms3.1215.

## Data Availability

The data that support the findings of this study are openly available in figshare at https://doi.org/10.6084/m9.figshare.19827940.v1.
